# In Silico Exploration of the Potential Role of Acetaminophen and Pesticides in the Etiology of Autism Spectrum Disorder

**DOI:** 10.3390/toxics9050097

**Published:** 2021-04-27

**Authors:** Tristan Furnary, Rolando Garcia-Milian, Zeyan Liew, Shannon Whirledge, Vasilis Vasiliou

**Affiliations:** 1 Environmental Health Sciences Department, Yale School of Public Health, New Haven, CT 06510, USA; tristan.furnary@yale.edu; 2 Bioinformatics Support Program, Cushing/Whitney Medical Library, Yale School of Medicine, New Haven, CT 06510, USA; rolando.milian@yale.edu; 3 Yale Center for Perinatal, Pediatric and Environmental Health, Yale School of Public Health, New Haven, CT 06510, USA; zeyan.liew@yale.edu; 4 Obstetrics, Gynecology & Reproductive Sciences, Yale School of Medicine, New Haven, CT 06510, USA; shannon.whirledge@yale.edu

**Keywords:** acetaminophen, pesticides, prenatal health, Autism Spectrum Disorder

## Abstract

Recent epidemiological studies suggest that prenatal exposure to acetaminophen (APAP) is associated with increased risk of Autism Spectrum Disorder (ASD), a neurodevelopmental disorder affecting 1 in 59 children in the US. Maternal and prenatal exposure to pesticides from food and environmental sources have also been implicated to affect fetal neurodevelopment. However, the underlying mechanisms for ASD are so far unknown, likely with complex and multifactorial etiology. The aim of this study was to explore the potential effects of APAP and pesticide exposure on development with regards to the etiology of ASD by highlighting common genes and biological pathways. Genes associated with APAP, pesticides, and ASD through human research were retrieved from molecular and biomedical literature databases. The interaction network of overlapping genetic associations was subjected to network topology analysis and functional annotation of the resulting clusters. These genes were over-represented in pathways and biological processes (FDR *p* < 0.05) related to apoptosis, metabolism of reactive oxygen species (ROS), and carbohydrate metabolism. Since these three biological processes are frequently implicated in ASD, our findings support the hypothesis that cell death processes and specific metabolic pathways, both of which appear to be targeted by APAP and pesticide exposure, may be involved in the etiology of ASD. This novel exposures-gene-disease database mining might inspire future work on understanding the biological underpinnings of various ASD risk factors.

## 1. Introduction

According to the Center for Disease Control and Prevention (CDC), 1 in 59 children born in 2008 in the United States have been diagnosed with Autism Spectrum Disorder (ASD), and the prevalence of ASD has increased in recent decades [1]. While broadening diagnosis criteria for autism is partially responsible for the observed increases in ASD prevalence, both genetic and environmental factors have also been suggested to contribute to ASD risk through gene-environment interactions [2,3].

Based on recent evidence, two prenatal exposures of importance to the risk of developing ASD are the common analgesic, APAP, and pesticides, which are highly present in our current environment and food [4]. APAP is the most commonly used drug during pregnancy, with approximately 65% of women taking the analgesic at some point during pregnancy [5]. This prenatal exposure has been associated with long-term negative effects on brain function [6]. Meanwhile, prenatal pesticide exposure, which likely increases with increased caloric intake during pregnancy, has been associated with traits related to ASD [7,8]. In fact, a recent systematic review found that among environmental toxicants, pesticides have one of the strongest associations with ASD risk [9,10].

Recent epidemiological cohort studies conducted in various countries have associated maternal use of APAP with increased risk of ASD and ASD-linked symptoms, such as internalizing and communication behavioral problems, in offspring and raise concerns that APAP may interfere with optimal fetal brain development [11,12,13,14,15,16]. For example, studies from the Danish National Birth Cohort have reported that maternal APAP used during pregnancy was associated with attention-deficit/hyperactivity disorder (ADHD) [11] and ASD cases co-morbid with hyperkinetic phenotypes [12]. A Spanish birth cohort study reported elevated childhood autism spectrum test scores among males prenatally exposed to APAP and stronger effect size with increasing frequency of maternal APAP use [13]. The Norwegian Mother and Child Cohort study used a same-sex sibling-pairs study design and revealed, through sibling-control analysis, that prenatal exposure to APAP for more than 28 days was associated with worse gross motor development, communication, externalizing and internalizing behaviors [14]. A recent study from the Boston Birth Cohort found concentrations of biomarkers for fetal exposure to APAP to be associated with significantly higher risk of ASD and ADHD [16]. Thus, the association between APAP exposure in utero and ASD is supported by consistent findings among studies. However, study limitations, including reliance on self-reported data, lack of information related to maternal exposure dose, and insufficient consideration of postnatal exposure, highlight the need for caution when assigning causation and underscore the necessity for studies that explore the mechanisms by which APAP exposure could contribute to the etiology of ASD. APAP can cross the placenta and the fetal brain barrier [17,18]. As such, APAP-induced oxidative stress, endocrine effects [19], and impacts on placental function [20] are suggested mechanisms that could influence fetal neurodevelopment.

Widespread environmental chemicals might modify the effects and toxicity of pharmaceutical agents, a concept known as drug-exposome interactions, resulting in reduced drug efficacy, increased drug resistance, or adverse effects [21,22]. Cross interactions between pollutants and drugs can potentially lead to inhibitory effects of drug metabolism, resulting in increased concentration of the drug in the body and amplification of pharmaceutical side effects [23]. The p-cresol metabolite produced by bacteria reduces the body’s ability to metabolize APAP, potentially increasing the risk of APAP-induced toxicity [24]. Environmental contaminants can also interact with various drug transporters, reduce drug-elimination rates, and thus increase the toxicity of the medication [25]. One instance of environmental exposures modifying the effectiveness of medication is the association between higher air pollutant exposure and lower risk of ASD among mothers consuming high levels of folic acid during the first month of pregnancy [26]. This concept has been proposed in the multicausal or multifactorial disease model, hypothesizing that a disease can be produced by various sufficient sets of genetic and environmental factors to induce disease occurrence [27].

Another possible environmental agent that could target similar pathways or modify the effects of APAP would be neurotoxic pesticide compounds, which have also been linked to ASD risk [9,28]. Some mechanistic studies have suggested that pesticide compounds that target receptors on the blood-brain barrier prevent drug transport and delivery through the brain, potentially leading to pharmaceutical ineffectiveness and other adverse effects [29]. One method by which pesticide exposure can alter or worsen the intended effect of medications is by interfering with the P-glycoprotein system, one of the body’s natural detoxification mechanisms found in secretory or barrier tissues such as the placenta and the vascular endothelial cells of the central nervous system [30]. However, there have been no studies that consider both APAP and pesticide exposures effects on human health.

The work presented here is a novel, first attempt to explore whether there are shared underlying biological pathways connecting genes associated with these two external exposure risk factors for the development of ASD. Both APAP and numerous pesticide compounds can function as oxidative stressors and subsequently affect neurodevelopment [6]. Although the mechanism by which oxidative stress may affect neurodevelopment and ASD is not fully understood, it is accepted that the nervous system is vulnerable to oxidative stress-mediated injury [31]. One proposed mechanism connecting oxidative stress with ASD implicates membrane lipid abnormalities, inflammation, impaired energy metabolism, and excitotoxicity as being involved in the pathogenesis of ASD [6,32].

Studies have shown that prenatal exposure to pesticides may result in the occurrence of general neurobehavioral disorders [33]. Of the 37 studies considered in one systematic review, 34 found an association between estimated environmental exposure to toxicants and ASD risk, with some of the strongest evidence implicating pesticides in ASD [9]. Ambient prenatal exposure to pesticides, such as glyphosate and chlorphyrifos, has also been associated with increased risk of ASD [28]. Although the external validity of some of these studies is limited to examining the effects of ambient, prenatal pesticide exposure in agricultural areas, they still offer insight to the potential effects of prenatal pesticide exposure among the general population, which is exposed via ingestion and residential use.

To explore whether APAP and pesticide exposure might influence ASD risk through similar underlying biological mechanisms, we conducted this study to perform database mining of the genetic associations of ASD in combination with network and functional analysis of candidate genes. Computer-based text mining methods have been used before to generate biomedical hypotheses on the impact of multiple exposures by examining novel associations between genes and diseases [34]. For example, literature mining and computational systems biology methods were used to explore the possible etiologic links between environmental chemicals, genes of interest, and type II diabetes [35]. By applying integrated molecular interaction network mining and text mining techniques, a previous study created a molecular connectivity map with novel information on AD-related genes and proteins to identify candidate drugs [36]. Such an in silico approach is particularly apt for addressing the present research question as it can simultaneously consider one specific exposure, APAP, with a broad class of exposures, pesticides, with regards to a clinically heterogeneous disease, ASD. The aim of the present study was to mine literature and molecular databases for genetic associations between APAP, pesticides, and ASD, which were then functionally annotated to lead to a novel perspective in understanding autism’s etiology. This work may shed light on the underlying biological mechanisms APAP and pesticide exposures have on ASD risk by targeting different genes on similar biological pathways and can offer guidance to future research on the pathogenesis of ASD.

## 2. Materials and Methods

Finding and retrieving genetics associations: Lists of genes associated with the exposures and disease of interest (APAP, Pesticides and ASD) were extracted from the public databases: Comparative Toxicogenomics Database (CTD) [37], PubChem [38], Open Targets Platform [39], DrugBank [40], PhegenI [41], and Clinvar [42], as well as from the proprietary knowledgebases Ingenuity Pathway Analysis (IPA) (QIAGEN Inc., Redwood City, CA, USA, https://www.qiagenbioinformatics.com/products/ingenuity-pathway-analysis, accessed in May 2020) [43] and MetaCore (Clarivate Analytics, Philadelphia, PA, USA). Appendix A provides a summary on each of these databases, while Appendix B indicates which databases were used to find and extract genes associated with each concept. 

The PubChem, DrugBank, CTD, Open Targets, and IPA databases were searched with keywords such as “Acetaminophen”, “Tylenol”, and “APAP” to identify genes in the human genome associated with APAP. Each database then matched the search term to existing concepts before executing the search. The search term “Tylenol”, for example, yielded results related to terms such as “Paracetamol”. For identifying genes associated with pesticides, the Chemicals and Diseases tabs were selected in independent searches of the term “Pesticides” using CTD. Each independent search of CTD employed different combinations of disease category, set to either Autism Disorder or Nervous System disease, and association type, set to curated or inferred. Finally, independent searches of the six molecular databases (Clinvar, PheGenI, MetaCore, IPA, Open Targets, and CTD) were performed to identify genes related to ASD using the search terms “Autism”, “Autism Spectrum Disorder”, or “Autism or Intellectual Disability” and find genes associated with ASD. The lists of genes generated by these searches were sorted into their respective categories (APAP, pesticides, ASD) and used to create Venn diagrams using InteractiVenn (http://www.interactivenn.net/, accessed in May 2020), which revealed overlapping genes between lists. The genes in each category that were identified in at least two databases, or the same database but with two independent search criteria, were used for further analysis.

To complement the results from the molecular databases, we used Coremine Medical (https://www.coremine.com/medical/, accessed in June 2020), a biomedical literature search engine, to retrieve genetic associations for APAP, pesticides and ASD. This is a mining tool that utilizes various ontologies (Medical Subject Headings, Gene Ontology) to map terms (e.g., diseases, genes) from the MEDLINE database. When any two terms are both found in any particular record, they become connected in the network. This tool also calculates a statistical score for the association between the two terms using a function that considers the frequency with which the entities co-occur versus their independent occurrence in the corpus as a whole (https://www.coremine.com/medical/help.html, accessed in June 2020). Genes significantly associated (*p* < 0.05) with the search terms (e.g., APAP, ASD) were used for further analysis. Both molecular database and literature methods are necessary as the former is more sensitive, while the latter is more specific to the search terms. Thus, incorporating both leads to more comprehensive results.

Pathway and network analysis: Ingenuity Pathway Analysis (Ingenuity Systems QIAGEN, Content version: 45865156, 2018, Redwood City, CA, USA) was used to carry out analyses for pathway, network, and molecular and cellular functions for found genetic associations. Each gene symbol was mapped onto its corresponding gene object in the Ingenuity Pathways Knowledge Base. This tool provides information about the overrepresentation (*p*-value of the Fisher’s Exact test and the corrected Benjamini-Hochberg FDR *p*-value) of these genes in pathways, diseases, and biological functions. We used the Comparison Analysis function in IPA to compare the pathways overrepresented in the genetic associations found in the molecular database and literature analyses. IPA was also used to find the associations of overlapping genes with neurodevelopment functions. This knowledgebase contains associations between molecules, diseases and functions manually curated from the biomedical literature. It also calculates the significance of the overlap using Fisher’s Exact test.

For network analysis, we used the STRING database to retrieve the gene interaction networks (https://string-db.org/, accessed in June 2020) database [44]—with a minimum required interaction score > 0.4. Cluster analysis of the resulting interaction networks was carried out using the MCODE application on the Cytoscape v3.8.0 software [45] with degree cutoff 2, node score cutoff 0.2, and K-core 2. Both IPA and STRING were used to reveal functional patterns emerging from the three search terms of interest.

## 3. Results

Six genes retrieved via molecular database mining were found in all three categories of interest: *ABCB1*, *ABCB4*, *CYP1A2*, *CYP3A4*, *FAS*, and *IGF1R*. These six genes were analyzed for ingenuity canonical pathways and then added to a functional network topological analysis along with their interacting genes, as determined by the STRING database. Carbohydrate metabolism and apoptosis were overrepresented as biological processes of common genes between APAP, pesticides and ASD risk. CTD produced the higher number of genetic associations. Sixteen different common genes to the three categories were found via literature mining using Coremine Medical: *CAT*, *CD4*, *TNF*, *VEGFA*, *EGFR*, *INS*, *AGT*, *IL2*, *FN1*, *PTH*, *TP53*, *ERBB2*, *GCG*, *TSC1*, *APP*, and *F2*. This approach was conducted to compliment the genetic associations found using molecular databases. These 16 genes were then used for pathway and network analysis. Carbohydrate transport, ROS metabolism, response to oxidative stress, growth, and regulation of ERK1 and ERK2 cascades as biological processes that relate to APAP, pesticides and ASD risk. Finally, comparison analysis of the two methods was done to reveal common findings.

### 3.1. Molecular Database Search for Genetic Associations

#### 3.1.1. Gene Retrieval from Molecular Databases

**Figure 1:**
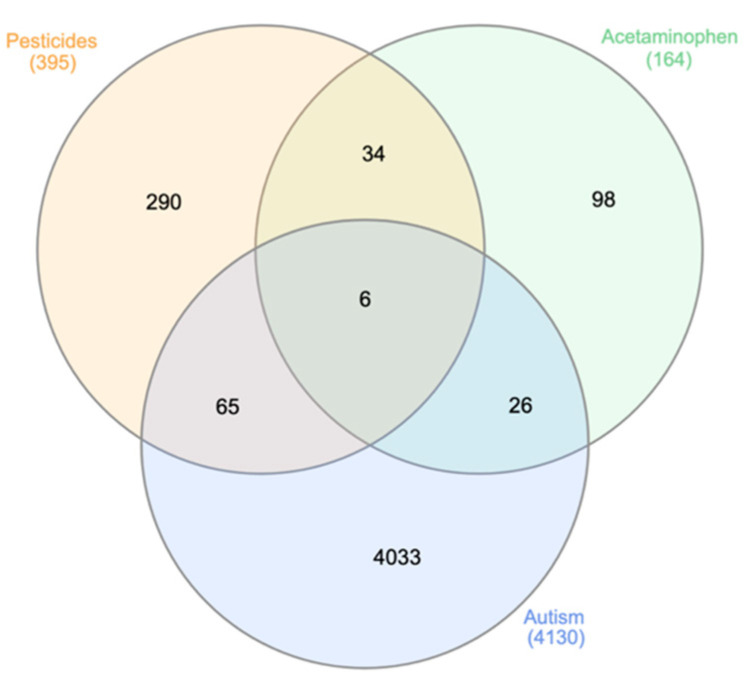
Venn diagram for overlapping genes associated with APAP, pesticides and ASD. The diagram shows the number of genes associated with APAP, pesticides, or ASD, or jointly with any two or all three factors. Six genes overlapped in all three categories: *ABCB1*, *ABCB4*, *CYP1A2*, *CYP3A4*, *FAS*, and *IGF1R*. These six genes were used for pathway analysis in IPA. The 65 genes found to overlap between the pesticide and ASD nodes, as well as the 26 genes overlapping between the APAP and ASD nodes, are listed in Appendix C.

The Venn diagram in Figure 1 contains the genes found in at least two separate searches from the molecular databases and shows that six genes overlap in the center: ATP Binding Cassette subfamily B member 1 and 4 (*ABCB1*, *ABCB4*), *CYP1A2*, *CYP3A4*, FAS Cell Surface Death Receptor (*FAS*), Insulin like Growth Factor Receptor 1 (*IGF1R*).

#### 3.1.2. Pathway Analysis

Table 1 presents the significant pathways overrepresented for the six common genes—identified from the database search—in the master Venn diagram (Figure 1). Each pathway is presented with Fisher’s Exact Test *p*-value of the overrepresentation analysis, and the overlapping genes. Results from this table are reassuring as pathways related to metabolism of foreign substances are present, as well as pathways that have been targeted in treatment efforts relating to ASD.

#### 3.1.3. Network Analysis

**Figure 2:**
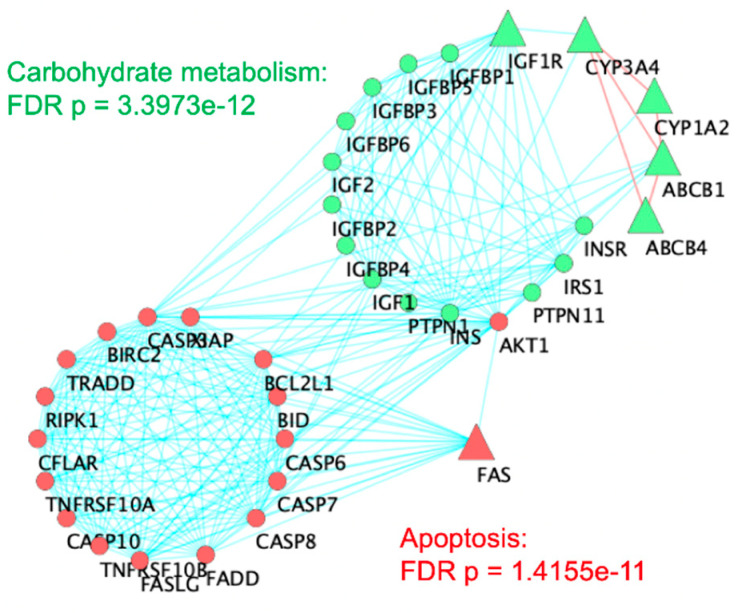
Network topological analysis of the six genes overlapping among ASD, pesticides, and acetaminophen. A cluster analysis of the STRING Protein–Protein Interaction network was carried out in Cytoscape. Triangles indicate the six original genes while circular nodes are first neighbors from STRING database interactions. Red lines connecting triangular nodes indicate connections between six original genes. Red nodes are overrepresented for apoptosis (FDR *p* = 1.41 × 10^−^^11^). Green nodes are overrepresented for carbohydrate metabolism (FDR *p* = 3.39 × 10^−^^12^).

The interaction network in Figure 2 shows the six genes from the master Venn diagram represented as large triangles and interacting genes from the STRING database as circles. Red nodes relate to apoptosis, or programmed cell death (FDR *p* = 1.41 × 10^−^^11^). Green nodes comprise a cluster that is significantly related to carbohydrate metabolism (FDR *p* = 3.39 × 10^−^^12^).

### 3.2. Literature Search for Genetic Associations

#### 3.2.1. Gene Retrieval from Coremine Medical

**Figure 3:**
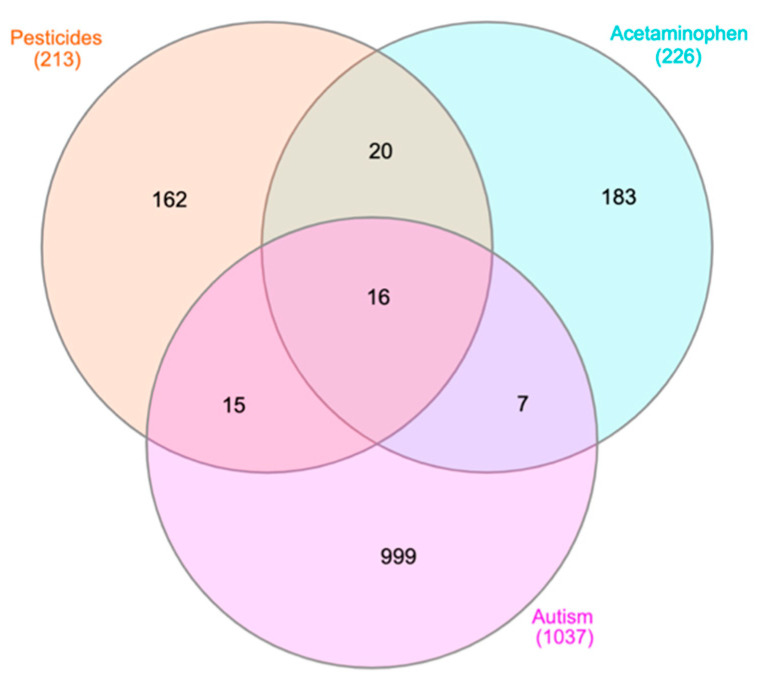
Venn diagram for genes retrieved from Coremine Medical associated with APAP, pesticides, and ASD. The 16 genes from the center of this Venn diagram are: *CAT*, *CD4*, *TNF*, *VEGFA*, *EGFR*, *INS*, *AGT*, *IL2*, *FN1*, *PTH*, *TP53*, *ERBB2*, *GCG*, *TSC1*, *APP*, and *F2*. These 16 genes are used in IPA to perform canonical pathway analysis, the results of which are shown in Table 2. The 15 genes found to overlap between the pesticide and ASD nodes, as well as the seven genes overlapping between the APAP and ASD nodes, are listed in Appendix E as they may be synergizing in common pathways.

In order to complement the results from different molecular databases, literature mining analysis was carried out using Coremine Medical. Figure 3 shows the results from Coremine in a Venn diagram of genes with significant (*p* < 0.05) frequencies of co-occurrence between the searched terms (e.g., ASD, APAP). 

#### 3.2.2. Pathway Analysis

The 16 genes found in the center node of Figure 3 are detailed in the discussion section. Ingenuity Pathway Analysis was used to assess the significance of the interactions between these 16 genes. Significant overrepresented functions include inositol lipid-mediated signaling (FDR *p* = 9.10 × 10^−^^7^), ERK1 and ERK2 cascade (FDR *p* = 9.20 × 10^−^^7^), and cell growth (FDR *p* = 1.40 × 10^−^^7^).

Over representation analysis using the STRING database of the 16 genes resulted in a protein–protein interaction enrichment *p*-value of 2.02 × 10^−^^7^. Such an enrichment *p*-value indicates that the proteins are at least partially biologically connected, as a group. STRING database analysis also showed interesting biological processes in the functional enrichment of the 16-gene network. Of note, regulation of phosphate metabolic process (FDR *p* = 3.19 × 10^−^^12^), regulation of cell death (FDR *p* = 6.25 × 10^−^^9^), regulation of ROS metabolic process (FDR *p* = 2.00 × 10^−^^9^), regulation of ERK1 and ERK2 cascade (FDR *p* = 1.02 × 10^−^^9^), and regulation of apoptotic process (FDR *p* = 4.76 × 10^−^^8^) were all processes related to the interactions between these genes.

IPA pathway analysis results are shown in Table 2 along with their *p*-values and respective genes. Some relevant pathways include Telomerase Signaling (*p* = 5.45 × 10^−^^5^), FXR/RXR activation (*p* = 8.86 × 10^−^^5^), Type I Diabetes Mellitus (*p* = 6.08 × 10^−^^5^), as well as several neurological pathways, such as Hypoxia-inducible factor 1α (HIF1α) signaling (*p* = 2.82 × 10^−^^3^), Agrin Interactions at Neuromuscular Junction (*p* = 1.39 × 10^−^^3^), Neuregulin Signaling (*p* = 2.04 × 10^−^^3^), and Neuroprotective Role of THOP1 in Alzheimer’s Disease (*p* = 3.02 × 10^−^^3^).

#### 3.2.3. Network Analysis

**Figure 4:**
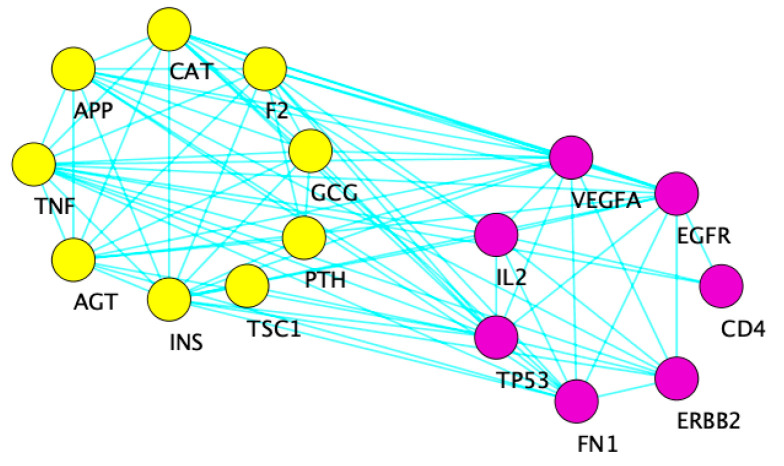
Cytoscape diagram showing the interaction network of 16 common genes between the three concepts from the Coremine search engine. The yellow cluster on the left is overrepresented for carbohydrate transport (FDR *p* = 1.39 × 10^−^^4^), ROS metabolic process (FDR *p* = 5.01 × 10^−^^5^), and response to oxidative stress (FDR *p* = 2.26 × 10^−^^4^). The right purple cluster is overrepresented for growth (FDR *p* = 2.98 × 10^−^^6^) and regulation of ERK1 and ERK2 cascade (FDR *p* = 1.08 × 10^−^^3^).

Figure 4 displays the interaction network resulting from the 16 overlapping genes among the three concepts from the Coremine Medical search engine. The cluster in yellow contains nine of the 16 genes, while the purple cluster contains the remaining seven.

### 3.3. Pathway Comparison Analysis

We compared the results from molecular datasets with those obtained with literature mining at the pathway level. Overrepresented common pathways are shown in Table 3. Aryl Hydrocarbon Receptor (AHR) signaling regulates xenobiotic metabolism and transposable elements that may control a large number of gene expression patterns [44]. Chemokine (C-C motif) Receptor 5 (CCR5) signaling in macrophages has been linked to neuroinflammation and neurodegeneration [46]. The above-mentioned FXR/RXR activation and those related to xenobiotic metabolism are also present in Table 3. Other pathways include PEDF signaling and PTEN signaling, which induces insulin resistance and modulates cell growth and apoptosis, respectively [47,48]. 

**Figure 5:**
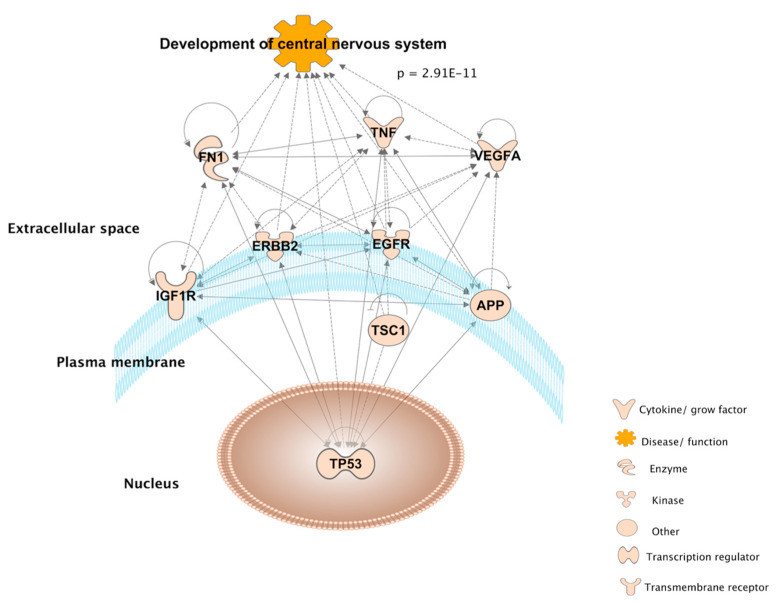
Ingenuity Pathway Analysis diagram connecting 9 out of the 22 overlapping genes from the molecular database and literature mining, as they are significantly associated with the development of the central nervous system according to Fisher’s Exact test (*p* = 2.91 × 10^−^^11^) in the IPA knowledgebase.

IPA knowledgebase showing the overlap between the 6 and 16 genes found via molecular database and literature mining approaches, respectively, as they relate to neurodevelopmental functions (Figure 5). Each arrow represents an association between molecules, diseases and functions manually curated from the biomedical literature. Nine out of 22 genes significantly overlap with the function Development of central nervous system, as calculated by the Fisher’s Exact test (*p* = 2.91 × 10^−^^11^), in the IPA knowledgebase.

## 4. Discussion

To the best of our knowledge, this is the first study to explore the potential effects of two common prenatal exposures, APAP and pesticides, on development with regards to the increasingly prevalent ASD. We found overlapping genes associated with APAP, pesticides, and ASD to be overrepresented in several biological pathways relating to apoptosis, metabolism of ROS, and carbohydrate metabolism. These pathways, many of which relate to the nervous system and xenobiotic metabolism, could elucidate the shared biology of APAP and pesticide exposures on ASD risk through the lens of genetic functions. Our results show that the considered co-exposures have many biological and mechanistic overlaps that connect them with the disease of interest and support the hypothesis that APAP and pesticides may target the same genes and molecular pathways.

The functions for each of the six genes uncovered through the molecular database associated with APAP, pesticides, and ASD, as well as the 16 genes elucidated through the literature mining approach, are presented in Table 4. Interestingly, there is no overlap between the individual genes selected by these two approaches. The molecular database and literature mining likely result in different genes because the former takes advantage of preexisting, published datasets, while the latter identifies significant genes associated with the exposures of interest in peer-reviewed studies. Ultimately, the two approaches aim to complement one another. Despite the absence of overlap between the two methods, the biological pathways elucidated via these two approaches have considerable amounts of significant overlap (Table 3). 

### 4.1. Main Biological Themes 

#### 4.1.1. Apoptosis

Various biological pathways identified here, such as ERK1 and ERK2 cascade, as well as telomerase signaling, play a direct role in the signaling pathway of apoptosis [50,51] and programmed cell death, which have been implicated in the etiology of ASD. Experiments have shown that 1 and 2 mM APAP concentrations, which are similar to the concentrations in human plasma during an APAP overdose, cause concentration-dependent neuronal death in vitro; additionally, APAP doses below what is required to induce acute hepatic failure (250 and 500 mg/kg) in vivo in rats lead to neuronal death [52]. CYPs in the brain have been suspected to play a role in this neurotoxic metabolism of APAP [53]. While neuronal cell death is clearly a consequence of high levels of APAP in cell and mouse models, one study found therapeutic doses of APAP to have no apparent toxic effects on human neuronal stem cells [54]. Still, the main limitation of this study is its model, which employs induced stem cells in vitro to infer the effects occurring in vivo. Thus, it is yet to be determined how APAP exposure effects developing human neurons in vivo, but prior studies have demonstrated effects on transformed neuronal stem cell lines in vitro utilizing toxic concentrations. HIF1α signaling (Table 2) has a role in the development of sympathetic ganglion neurons and is known to regulate genes that are involved in adaptive and protective neuronal processes during maturation [55,56]. In mice, genetic deletion of *HIF1α* leads to increased cell death and decreased proliferation of neuronal progenitor cells within the sympathetic nervous system [55]. Another mouse model also showed that inhibition or deletion of neuronal *HIF1α* increased necrotic and apoptotic cell death [56]. After exposing pregnant rats to glyphosate-based herbicides, one study found offspring to exhibit significant increases (73%, *p* = 0.017) in HIF1α in the cerebellum [57]. Following pesticide exposure, the placentas of a cohort of pregnant tea garden workers showed increased levels of *HIF1α* expression [58]. Neuropathological evidence suggests that activation of apoptosis during development may mechanistically explain the pathophysiology of ASD [59]. In mice treated with 300 mg/kg of APAP, *HIF1α* was induced even prior to the onset of any hepatoxic effects and contributed to oxidative stress, which also links it to ROS metabolism [60]. Thus, alterations in HIF1a expression may be an important contributing factor to the development of ASD. Future studies should address the effect of cumulative APAP and pesticide exposure on the regulation of HIF1a, as it is currently unclear whether APAP or APAP in combination with pesticides can alter *HIF1a* expression in a manner that would result in the activation of apoptosis.

An established connection between ROS and apoptosis is further supported by our finding of AHR signaling. AHR is a ligand-induced receptor involved in interactions between an individual and its environment [44]. After certain exposures, AHR signaling has been found to mediate apoptosis and neurotoxicity [61]. A study using mouse primary neuronal cells derived from neocortical and hippocampal tissues has shown that AHR activation by beta-naphthoflavone, an AHR agonist, leads to apoptosis [62]. Furthermore, AHR signaling is also connected with the metabolism of ROS via kynurenine, a ligand of AHR, which has been shown to stimulate AHR in human embryonic stem cells, which leads to increased expression of self-renewal genes [63]. It has been shown that microbiome-mediated AHR-ligands engage the host immune system and stimulate metabolic responses [64]. It is worth noting that many AHR ligands are able to cross the blood–brain barrier, implying a possible role of AHR in the central nervous system [64]. Therefore, it is possible that AHR receptor signaling, which is linked to APAP and pesticides, could participate in the etiology of ASD. Another apparent link between apoptosis and ROS metabolism is indicated by identification of the various xenobiotic, p53, and PTEN signaling pathways listed in Table 3.

#### 4.1.2. Metabolism of ROS

ROS metabolism has been implicated in perinatal mechanisms potentially leading to ASD’s clinical symptoms and its pathogenesis [6,31,32,65]. Excess production of ROS can impair DNA methylation, which normally silences certain genes, leading to a positive feedback mechanism that makes individuals with ASD more vulnerable to oxidative stress and neuronal toxicity [31]. It has also been speculated through an age- and sex group-matched case control study that different perinatal oxidative stress-related environmental and genetic factors could lead to the development of ASD [65]. Toxicity of APAP is mainly due the formation of APAP’s reactive intermediate, NAPQI, which leads to excessive formation of ROS [66,67]. In human neuroblastoma cells, exposure to the pesticides endosulfan and zineb resulted in the production of ROS in a dose- and time-dependent manner [68]. As suggested by the overlapping results presented here, it is possible that pesticides activate pathways that also lead to greater production of ROS, which would then place further ROS-stress on cells of interest. Another step in the mechanism by which APAP enacts its toxicity on cells is through glutathione (GSH) depletion [66]. GSH is the antioxidant involved in anti-ROS defense mechanisms [32]. APAP has been shown to significantly reduce GSH levels in the brains of mice [69]. Interestingly, GSH levels have been shown to be significantly lower in the cerebellum and Brodmann Area 22 (a portion of Wernicke’s area and thus assists in language comprehension) of individuals with ASD [70]. While the understanding of fetal metabolism of APAP as well as the safe reference level for fetal brain exposure to APAP and NAPQI are unclear, the metabolism of ROS is another biological process that connects co-exposure to APAP and pesticides with ASD. The combined risk that APAP and pesticides pose to normal ROS metabolism could have relevant implications in the etiology of ASD. A connection between ROS metabolism and carbohydrate metabolism has also been implicated in the pathogenesis of ASD [71].

#### 4.1.3. Carbohydrate Metabolism

Proper metabolism of carbohydrates is essential for normal neurodevelopment in order to supply the developing brain with the necessary energy demands needed for such dramatic changes in brain function and structure [72,73]. Studies have shown that impairments in carbohydrate metabolism in the brain are associated with ASD [71,74]. It is within reason that this study implicates carbohydrate metabolism in ASD’s etiology, considering that some pesticides exact their neurotoxic effects by targeting carbohydrate metabolism [75,76]. FXR/RXR activation and Type I Diabetes Mellitus (Table 3) are two pathways identified here that support this carbohydrate metabolism hypothesis. FXR/RXR activation regulates the metabolism of several carbohydrates, such as cholesterol, triglyceride, and glucose [77,78]. Type I Diabetes Mellitus relates to carbohydrate metabolism, as this disease’s predominant symptom is a decrease in the production of insulin. The sirtuin signaling pathway (Table 2) has been connected to the insulin/insulin-like growth factor 1 signaling pathway [79]. Thus, it is also associated with the *IGF1R* (Figure 2), which has been connected to ASD via neo-neuron myelination [80]. 

### 4.2. Intra-Pathway Interactions

APAP and pesticides may not always affect the same genes, apart from those identified in the center of the presented Venn diagrams, but they are associated with genes related to the same pathways, which is relevant because no single gene or region of the genome is responsible for ASD [81]. If the malfunctioning of a pathway is implicated in the etiology of ASD, then affecting multiple genes related to that pathway is more likely to increase the risk of developing ASD. If one exposure is affecting several genes on a given pathway, and the other is also affecting genes on that same pathway, their combined effect could lead to a loss of function or an upregulation of that pathway. Existing research supports a potential multiplicative association between APAP and pesticides in relation to ASD, as both APAP and many pesticides show a significant degree of bias towards, or selective targeting of, autism susceptibility genes [82]. One illustration of this is demonstrated when considering the Reelin signaling pathway. Mutations in the RXR motif suggested in our pathway analyses are highly associated with the development of ASD; however, these mutations on their own are insufficient and likely secondary genetic or environmental factors for a diagnosis [83]. Interestingly, mammalian target of rapamycin (mTOR) signaling is also implicated in this study as the downstream signaling of the reelin pathway interacts with the mTOR pathway [83]. While the exact effect (e.g., upregulation, downregulation, silencing) these exposures have on certain pathways is not ascertainable through the methods employed here, the overlap presented here in itself suggests the potential for pesticides and APAP to modify the adverse exposure effects of one another. Altogether, co-exposure to APAP and pesticides may be involved in the still unknown etiology of ASD by route of the pathways identified here and should be considered in future studies. A multitude of other anthropogenic chemicals, such as PCBs, air pollutants, heavy metals, and phthalates [9], may also interact in a manner that leads to suboptimal neurodevelopment, but given the genetic and biological overlap presented here, as well as the commonness of the considered exposures, attention should be given to APAP and pesticide exposures in conjunction. Future studies could also expand this in silico analytical approach to include the aforementioned environmental pollutants that have been linked to ASD risk.

### 4.3. Strengths and Limitations

This novel network analysis reveals patterns that add to the evidence in favor of studying a mixture of APAP and pesticides in relation to ASD. A major strength of this study’s exploratory, hypothesis-generating approach is the ability of in silico methods to relate and consolidate genetic associations of APAP, a widely used medication in pregnancy, with pesticides, a broad class of chemicals, and with ASD, a heterogeneous disorder with varying impacts on the lives of affected individuals. The use of broad inclusion criteria for pesticides in the methods may appear too heterogeneous; however, because pesticide exposures among pregnant women are common and variable, a strength of the present approach is the general inclusion criteria that capture all pesticide exposures, which might vary by location, age, occupation and socioeconomic status. One limitation of this work is its reliance on public databases. The quality of such databases cannot always be controlled because the extent of their reporting excludes effect size and effect direction, and they lack comprehensive lists of genetic associations with pesticides. Additionally, these methods do not enable us to differentiate between exposure timing. Thus, database gene-exposure associations are not specific to perinatal exposure but instead represent associations among the adult population; however, a literature review reveals that the 22 genes listed in Table 4 are discussed to some extent with consideration of ASD and pre- and perinatal neurodevelopment in 35 studies. These studies include several rodent models, epigenetic approaches, and case studies, and consider various environmental exposures. We set a threshold of two to avoid false positive signals in our network analyses, but this could have resulted in a small degree of overlap within factors, thus missing some important genetic information or pathways. 

APAP and pesticides are widespread exposures that have been implicated to affect risk of ASD, but the exposure-disease mechanisms were unknown. We presented a novel in silico analysis by mining literature and molecular databases to elucidate the potential role of these exposures in the etiology of ASD. Our findings suggest apoptosis, ROS metabolism, and carbohydrate metabolism as biological pathways intertwined in a possible mechanism of ASD’s etiology. Further, our results reveal the potential for intra-pathway interactions regarding genes and biological processes as a possible means by which APAP and pesticide co-exposure may modify the adverse exposure effects of one another. Future biological tests are needed to show the interactions between these genes of interest as they relate to the considered exposures and their larger roles in affecting apoptosis, the metabolism of ROS, and carbohydrate metabolism during development. Such work would involve in vivo or in vitro models and should consider testing the exposure mixtures of APAP and select pesticides that have been shown to affect neurodevelopment via developmental neurotoxicity studies and are evidenced to target the three biological processes identified here.

## Figures and Tables

**Table 1:** Ingenuity Pathway Analysis showing overrepresented pathways (Fisher’s Exact Test *p* < 0.05) of the six genes found in the molecular databases. Pathways with significant *p*-values > 0.01 are displayed in Appendix D.

Ingenuity Canonical Pathways	*p*-Value	Genes
PXR/RXR Activation	4.47 × 10^−^^7^	*ABCB1*, *CYP1A2*, *CYP3A4*
Xenobiotic Metabolism CAR Signaling Pathway	1.12 × 10^−^^5^	*ABCB1*, *CYP1A2*, *CYP3A4*
Bupropion Degradation	1.74 × 10^−^^5^	*CYP1A2*, *CYP3A4*
Acetone Degradation I (to Methylglyoxal)	2.51 × 10^−^^5^	*CYP1A2*, *CYP3A4*
Xenobiotic Metabolism Signaling	3.89 × 10^−^^5^	*ABCB1*, *CYP1A2*, *CYP3A4*
Estrogen Biosynthesis	4.79 × 10^−^^5^	*CYP1A2*, *CYP3A4*
Aryl Hydrocarbon Receptor Signaling	5.75 × 10^−^^4^	*CYP1A2*, *FAS*
Hepatic Fibrosis/Hepatic Stellate Cell Activation	9.77 × 10^−^^4^	*FAS*, *IGF1R*
Xenobiotic Metabolism PXR Signaling Pathway	1.05 × 10^−^^3^	*ABCB1*, *CYP3A4*
LPS/IL-1 Mediated Inhibition of RXR Function	1.41 × 10^−^^3^	*ABCB1*, *CYP3A4*

**Table 2:** Ingenuity pathway analysis showing significant (Fisher’s Exact Test *p* < 0.05) pathways overrepresented for the 16 genes found via Coremine Medical. Pathways with significant *p*-values > 0.01 are displayed in Appendix F.

Ingenuity Canonical Pathways	*p*-Value	Genes
Acute Phase Response Signaling	6.306 × 10^−^^6^	*AGT*, *F2*, *FN1*, *TNF*
Hepatic Cholestasis	7.244 × 10^−^^6^	*GCG*, *IL2*, *INS*, *TNF*
Telomerase Signaling	5.494 × 10^−^^5^	*EGFR*, *IL2*, *TP53*
Type I Diabetes Mellitus Signaling	6.026 × 10^−^^5^	*IL2*, *INS*, *TNF*
Estrogen Receptor Signaling	6.768 × 10^−^^5^	*AGT*, *EGFR*, *TP53*, *VEGFA*
FXR/RXR Activation	8.91 × 10^−^^5^	*AGT*, *INS*, *TNF*
NF-κB Signaling	2.51 × 10^−^^4^	*EGFR*, *INS*, *TNF*
mTOR Signaling	3.98 × 10^−^^4^	*INS*, *TSC1*, *VEGFA*
Hematopoiesis from Pluripotent Stem Cells	5.37 × 10^−^^4^	*CD4*, *IL2*
Myc Mediated Apoptosis Signaling	5.62 × 10^−^^4^	*TNF*, *TP53*
PXR/RXR Activation	9.33 × 10^−^^4^	*INS*, *TNF*
Sirtuin Signaling Pathway	1.02 × 10^−^^3^	*APP*, *TNF*, *TP53*
VDR/RXR Activation	1.35 × 10^−^^3^	*IL2*, *PTH*
Agrin Interactions at Neuromuscular Junction	1.39 × 10^−^^3^	*EGFR*, *ERBB2*
Glucocorticoid Receptor Signaling	1.55 × 10^-3^	*AGT*, *IL2*, *TNF*
Allograft Rejection Signaling	1.62 × 10^−^^3^	*IL2*, *TNF*
Crosstalk between Dendritic Cells and Natural Killer Cells	1.78 × 10^−^^3^	*IL2*, *TNF*
OX40 Signaling Pathway	1.82 × 10^-3^	*CD4*, *IL2*
ErbB Signaling	1.95 × 10^−^^3^	*EGFR*, *ERBB2*
Neuregulin Signaling	2.04 × 10^−^^3^	*EGFR*, *ERBB2*
Apoptosis Signaling	2.19 × 10^−^^3^	*TNF*, *TP53*
PPAR Signaling	2.40 × 10^−^^3^	*INS*, *TNF*
HIF1α Signaling	2.82 × 10^−^^3^	*TP53*, *VEGFA*
Neuroprotective Role of THOP1 in Alzheimer’s Disease	3.02 × 10^−^^3^	*AGT*, *APP*
Insulin Receptor Signaling	4.27 × 10^−^^3^	*INS*, *TSC1*
Type II Diabetes Mellitus Signaling	4.37 × 10^−^^3^	*INS*, *TNF*
Necroptosis Signaling Pathway	5.37 × 10^−^^3^	*TNF*, *TP53*
Mitochondrial Dysfunction	6.31 × 10^−^^3^	*APP*, *CAT*
PI3K/AKT Signaling	6.61 × 10^−^^3^	*TP53*, *TSC1*

**Table 3:** Pathway comparison analysis. Table shows significant pathways found amongst all 22 genes retrieved through molecular databases and literature mining. Rows sorted by alphabetical order of canonical pathway.

Canonical Pathways	Molecular Database Analysis *p*-Value	Literature Analysis *p*-Value
Aryl Hydrocarbon Receptor Signaling	9.79 × 10^−^^4^	8.86 × 10^−^^5^
CCR5 Signaling in Macrophages	2.69 × 10^−^^2^	1.69 × 10^−^^2^
Death Receptor Signaling	2.87 × 10^−^^2^	1.85 × 10^−^^2^
FXR/RXR Activation	2.25 × 10^−^^2^	4.46 × 10^−^^3^
LPS/IL-1 Mediated Inhibition of RXR Function	9.69 × 10^−^^4^	6.78 × 10^−^^5^
Myc Mediated Apoptosis Signaling	1.29 × 10^−^^2^	5.59 × 10^−^^4^
NF-κB Signaling	1.60 × 10^−^^2^	1.76 × 10^−^^3^
p53 Signaling	3.05 × 10^−^^2^	4.01 × 10^−^^2^
PEDF Signaling	2.59 × 10^−^^2^	1.18 × 10^−^^2^
PTEN Signaling	2.38 × 10^−^^2^	1.05 × 10^−^^2^
PXR/RXR Activation	4.45 × 10^−^^7^	9.43 × 10^−^^4^
Type I Diabetes Mellitus Signaling	2.15 × 10^−^^2^	2.17 × 10^−^^3^
Xenobiotic Metabolism CAR Signaling Pathway	1.40 × 10^−^^3^	1.97 × 10^−^^4^
Xenobiotic Metabolism PXR Signaling Pathway	5.81 × 10^−^^4^	6.08 × 10^−^^5^
Xenobiotic Metabolism Signaling	1.04 × 10^−^^3^	1.50 × 10^−^^4^

**Table 4:** Resulting genetic associations for the interaction between APAP, pesticides and ASD.

Source of Association	Official Gene Symbol	Name	NCBI Gene Description/Function
Molecular databases	*ABCB1*	ATP Binding Cassette subfamily B member 1	multidrug resistance, and ATP-dependent drug efflux pumps for xenobiotic compounds; transporter in the blood-brain barrier
	*ABCB4*	ATP Binding Cassette subfamily B member 4	
	*CYP1A2*	Cytochrome P450 Family 1 Subfamily A Member 2	catalyzes many reactions involved in drug metabolism and the synthesis of cholesterol, steroids and other lipids
	*CYP3A4*	Cytochrome P450 Family 3 Subfamily A Member 4	metabolizes steroids as well as carcinogens, involved in the metabolism of approximately half of all drugs currently in use
	*FAS*	Fas Cell Surface Death Receptor	contains a death domain, plays a central role in the physiological regulation of programmed cell death, involved in transducing the proliferating signals in normal diploid fibroblast and T cells
	*IGF1R*	Insulin like Growth Factor Receptor 1	binds insulin-like growth factor, highly overexpressed in most malignant tissues, functions as anti-apoptotic agent by enhancing cell survival
Literature	*CAT*	Catalase	enzyme that protects cells from ROS-induced oxidative damage
	*CD4*	Cluster of differentiation 4	membrane glycoprotein of T lymphocytes
	*TNF*	Tumor Necrosis Factor	triggers activation of the MLKL cascade which is critical in the generation of ROS
	*VEGFA*	Vascular Endothelial Growth Factor A	encodes heparin-binding protein, induces proliferation and migration of vascular endothelial cells
	*EGFR*	Epidermal Growth Factor Receptor	encodes for a transmembrane glycoprotein that is a member of the protein kinase superfamily
	*INS*	Insulin	peptide hormone, plays major role in regulating carbohydrate and lipid metabolism
	*AGT*	Angiotensinogen	codes for a liver protein involved in maintaining blood pressure
	IL2 *	Interleukin 2	encodes for a protein important in the proliferation of B and T lymphocytes
	*FN1*	Fibronectin 1	codes for fibronectin, a protein involved in cell adhesion and migration processes including embryogenesis
	*PTH*	Parathyroid Hormone	encodes a preproprotein that is proteolytically processed to a protein involved in parathyroid hormone signaling
	*TP53*	Tumor Protein p53	tumor repressor protein involved in cellular stress responses that can induce cell cycle arrest, apoptosis, senescence, DNA repair, and changes in metabolism
	*ERBB2*	erb-b2 Receptor Tyrosine Kinase 2	encodes a member of the epidermal growth factor receptor family
	*GCG*	Glucagon	stimulates glycogenolysis and gluconeogenesis
	*TSC1*	TSC Complex Subunit 1	encodes hamartin, a growth inhibitory protein
	*APP*	Amyloid Beta Precursor Protein	serves as a cell surface receptor and transmembrane protein that is cleaved to form several types of peptides
	*F2*	Coagulation Factor II, Thrombin	encodes the prothrombin protein that is cleaved in several steps to generate thrombin, a protein that plays a role in cell proliferation, tissue repair, and maintaining vasculature during perinatal development

* Interestingly, *IL2*′s related gene, interleukin 1β (*IL1β*), was found to be significantly increased in the plasma of fetuses taken from pregnant rats treated with APAP [49].

**Figure A1:**
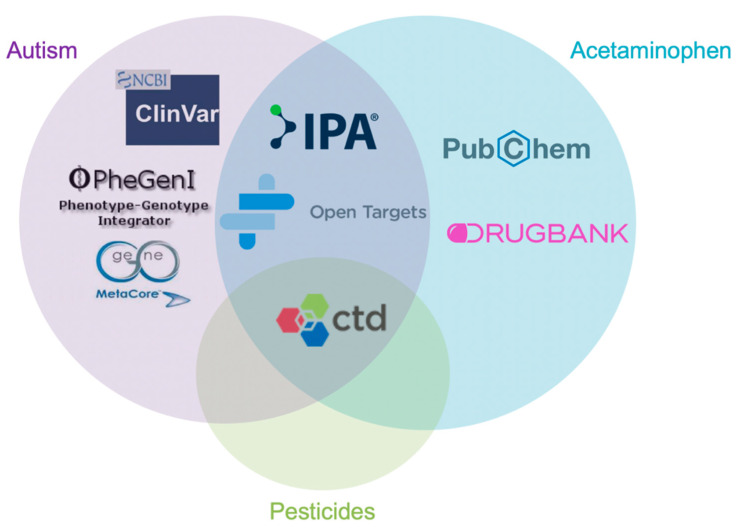
Data acquisition of lists of candidate genes were taken from various molecular databases. Databases include Comparative Toxicogenomics Database, Pubchem, Open Targets Platform, Ingenuity Pathway Analysis, DrugBank, PhegenI, MetaCore, and Clinvar. Each of these databases was used for one or more of the three factors, these being acetaminophen, pesticides and or autism.

## Data Availability

Lists of genes associated with the exposures and disease of interest were extracted from the public databases, as well as the proprietary knowledgebases are listed in Appendix A.

## Appendix A

**Table A1:** Table describing each database used for network topological analysis work. Information taken from the about page of each database’s website.

Database	Information about Database
CTD	CTD is a robust, publicly available database that aims to advance understanding about how environmental exposures affect human health. It provides manually curated information about chemical–gene/protein interactions, chemical–disease and gene–disease relationships. These data are integrated with functional and pathway data to aid in development of hypotheses about the mechanisms underlying environmentally influenced diseases.
PubChem	PubChem is an open chemistry database at the National Institutes of Health (NIH). “Open” means that you can put your scientific data in PubChem and that others may use it. Since the launch in 2004, PubChem has become a key chemical information resource for scientists, students, and the general public. Each month our website and programmatic services provide data to several million users worldwide.
Open Targets Platform	The Open Targets Platform is a comprehensive and robust data integration for access to and visualization of potential drug targets associated with disease. It brings together multiple data types and aims to assist users to identify and prioritize targets for further investigation.
IPA	IPA is an all-in-one, web-based software application that enables analysis, integration, and understanding of data from gene expression, miRNA, and SNP microarrays, as well as metabolomics, proteomics, and RNAseq experiments. IPA can also be used for analysis of small-scale experiments that generate gene and chemical lists. IPA allows searches for targeted information on genes, proteins, chemicals, and drugs, and building of interactive models of experimental systems. Data analysis and search capabilities help in understanding the significance of data, specific targets, or candidate biomarkers in the context of larger biological or chemical systems. The software is backed by the Ingenuity Knowledge Base of highly structured, detail-rich biological and chemical findings.
DrugBank	The DrugBank database is a comprehensive, freely accessible, online database containing information on drugs and drug targets. As both a bioinformatics and a cheminformatics resource, DrugBank combines detailed drug (i.e., chemical, pharmacological and pharmaceutical) data with comprehensive drug target (i.e., sequence, structure, and pathway) information. Because of its broad scope, comprehensive referencing and unusually detailed data descriptions, DrugBank is more akin to a drug encyclopedia than a drug database. As a result, links to DrugBank are maintained for nearly all drugs listed in Wikipedia. DrugBank is widely used by the drug industry, medicinal chemists, pharmacists, physicians, students and the general public. Its extensive drug and drug-target data has enabled the discovery and repurposing of a number of existing drugs to treat rare and newly identified illnesses.
PheGenI	The Phenotype-Genotype Integrator (PheGenI), merges NHGRI genome-wide association study (GWAS) catalog data with several databases housed at the National Center for Biotechnology Information (NCBI), including Gene, dbGaP, OMIM, eQTL and dbSNP
MetaCore	MetaCore provides the core capabilities of precise pathway analysis, knowledge mining, simple bioinformatics and effective visualizations in a comprehensive, off-the-shelf package. Use high-quality, 100% manually curated biological pathway data from peer-reviewed literature to accelerate drug development by rapidly generating and validating hypotheses for novel biomarkers, targets and mechanisms of action.
Clinvar	ClinVar processes submissions reporting variants found in patient samples, assertions made regarding their clinical significance, information about the submitter, and other supporting data. The alleles described in submissions are mapped to reference sequences, and reported according to the HGVS standard
CoreMine Medical	Coremine Medical™, the first domain-specific information community built on top of the COREMINE Platform. It is a free Internet service for searching, updating, and sharing medical information–both search and social network.

## Appendix C

The 65 genes found to overlap between the pesticide and ASD nodes of the molecular database analysis in Figure 1 are: *PON1*, *ACHE*, *GSTM1*, *BDNF*, *CYP2D6*, *MTHFR*, *TH*, *CYP19A1*, *ESR1*, *AR*, *PRDX6*, *CYP11A1*, *GRIN1*, *LEP*, *NECAP1*, *SERPINE1*, *CAPN3*, *CCL3*, *CHCHD2*, *FAH*, *LAMB1*, *LAMP2*, *NTF3*, *PPARA*, *RASGRF1*, *SC5D*, *SERPINC1*, *CYP1A1*, *PRDX4*, *FOXO1*, *G6PD*, *MID1IP1*, *ACACA*, *C1R*, *CD36*, *CREB5*, *DGKK*, *EGFR*, *FABP4*, *GAPDH*, *LHX1*, *MIOX*, *NBPF10*, *NBPF11*, *NBPF12*, *NBPF15*, *NBPF8*, *NBPF9*, *NEFM*, *NFE2L3*, *NHS*, *NOTCH2NLA*, *OLR1*, *OPCML*, *PAIP2B*, *PIR*, *PKM*, *PTPN9*, *SERPINF2*, *SNORA5A*, *SOX9*, *TPT1*, *UGP2*, *WNT7B*, *ZNF202*.

The 26 genes that overlap between the APAP and ASD nodes of the molecular database analysis in Figure 1 are: *UGT1A6*, *IL6*, *AIFM1*, *PRKN*, *RELA*, *ABCC1*, *IFRD1*, *IGFBP1*, *GJB1*, *SORD*, *ACTB*, *CYCS*, *NDRG2*, *SERPINF1*, *CAST*, *LIPC*, *PRKCB*, *ACO1*, *ASL*, *CCNG1*, *CYP3A5*, *IGFBP3*, *CXCR3*, *NR0B1*, *TUBB*, *UGT1A9*.

## Appendix D

**Table A2:** Ingenuity Pathway Analysis showing significant (Fisher’s Exact Test *p* ≤ 0.05) pathways related to the 6 genes found in the molecular databases. These significant pathways are associated with *p*-values > 0.01.

Ingenuity Canonical Pathways	*p*-Value	Genes
Death Receptor Signaling	2.40 × 10^−^^2^	*FAS*
p53 Signaling	2.57 × 10^−^^2^	*FAS*
Apoptosis Signaling	2.57 × 10^−^^2^	*FAS*
IGF-1 Signaling	2.69 × 10^−^^2^	*IGF1R*
Type I Diabetes Mellitus Signaling	2.88 × 10^−^^2^	*FAS*
FXR/RXR Activation	3.31 × 10^−^^2^	*ABCB4*
Necroptosis Signaling Pathway	4.07 × 10^−^^2^	*FAS*

## Appendix E

The 15 genes found to overlap between the pesticide and ASD nodes of the literature analysis are: *LRRC4C*, *BMND7*, *BMND8*, *MS*, *CRP*, *SLC18A2*, *PLG*, *IFNG*, *NFKB1*, *ARCN1*, *COPD*, *KLK3*, *MYC*, *IL1B*, *AVP*. The 7 genes that overlap between the APAP and ASD nodes of the literature analysis are: *ALB*, *FBXO15*, *CASP3*, *CNR1*, *MMD2*, *MIR124-1*, *CYCS*.

## Appendix F

**Table A3:** Ingenuity Pathway Analysis showing significant (Fisher’s Exact Test *p* < 0.05) related to the 16 genes found using Coremine Medical as a text search engine. These significant pathways are associated with *p*-values > 0.01.

Ingenuity Canonical Pathways	*p*-Value	Genes
EIF2 Signaling	1.07 × 10^−^^2^	*INS*, *VEGFA*
Huntington’s Disease Signaling	1.17 × 10^−^^2^	*EGFR*, *TP53*
Senescence Pathway	1.58 × 10^−^^2^	*CAT*, *TP53*
Xenobiotic Metabolism Signaling	1.70 × 10^−^^2^	*CAT*, *TNF*
Neuroinflammation Signaling Pathway	1.86 × 10^−^^2^	*APP*, *TNF*
Axonal Guidance Signaling	4.47 × 10^−^^2^	*ERBB2*, *VEGFA*
